# Scores to predict steatotic liver disease – correlates and outcomes in older adults

**DOI:** 10.1038/s44355-025-00021-3

**Published:** 2025-04-19

**Authors:** Daniel Clayton-Chubb, Isabella Commins, Stuart K. Roberts, Ammar Majeed, Robyn L. Woods, Joanne Ryan, Hans G. Schneider, John S. Lubel, Alexander D. Hodge, John J. McNeil, William W. Kemp

**Affiliations:** 1 Department of Gastroenterology, Alfred Health, Melbourne, VIC Australia; 2 School of Translational Medicine, Monash University, Melbourne, VIC Australia; 3 Department of Gastroenterology, Eastern Health, Melbourne, VIC Australia; 4 Department of Gastroenterology, St Vincent’s Hospital, Melbourne, VIC Australia; 5 School of Public Health and Preventive Medicine, Monash University, Melbourne, VIC Australia; 6 Department of Pathology, Alfred Health, Melbourne, VIC Australia; 7 Department of Gastroenterology, Northern Health, Melbourne, VIC Australia; 8 School of Health and Biomedical Science, RMIT University, Melbourne, VIC Australia; 9 Department of Medicine, Eastern Clinical School, Monash University, Melbourne, VIC Australia

**Keywords:** Liver, Liver diseases, Predictive markers, Diagnosis

## Abstract

Metabolic dysfunction-associated steatotic liver disease (MASLD) is a significant cause of chronic liver disease globally, and the rising prevalence of MASLD is occurring in parallel with the global aging population. The use of non-invasive biomarker tools to rule-in or rule-out hepatic steatosis is important in large epidemiological studies in this field. While the Fatty Liver Index (FLI) is the best validated tool in older adults, not all studies will have the necessary parameters for steatosis identification. This retrospective post-hoc analysis of the ASPirin in Reducing Events in the Elderly (ASPREE) study involved 16,703 Australian adults aged ≥70 years. Using the FLI as the ‘gold standard’ index, we evaluated the correlation with other indices: the Dallas Steatosis Index (DSI), Framingham Steatosis Index, ZJU index (ZJU), Hepatic Steatosis Index (HSI), Lipid Accumulation Product (LAP), and Visceral Adiposity Index (VAI), as well as age- and sex-adjusted outcome measures including mortality, major adverse cardiovascular events (MACE), atrial fibrillation (AF), and persistent physical disability. Of the non-FLI indices, the DSI and FSI had the highest percentage of participants correctly classified as having MASLD (97.7% and 93.8% respectively). The FSI, LAP, and VAI were associated with MACE. The FSI and FLI were predictive of incident AF. The FLI, DSI, FSI, LAP and VAI were associated with physical disability. No MASLD score was associated with increased mortality. Indeed, MASLD defined by the ZJU and HSI were both inversely associated with mortality. As such, we’ve demonstrated that the FSI and DSI are the most accurate scores for identifying MASLD in older adults when compared to the FLI as the gold standard. The FSI is associated with MACE, AF, and persistent physical disability, lending support to its use in identifying older persons with MASLD when the FLI is unable to be calculated.

## Introduction

Metabolic dysfunction-associated steatotic liver disease (MASLD)^1^ is a chronic liver disease estimated to affect up to 38% of the global population^2^. Its prevalence is increasing in conjunction with increasing rates of obesity, type 2 diabetes mellitus (T2DM), and other cardio-metabolic comorbidities. MASLD may progress to cirrhosis and hepatocellular carcinoma, and has also been associated with increased rates of cardiovascular disease^3^, sarcopenia^4^, and in some studies mortality^5^.

Rising in tandem with the increasing prevalence of MASLD is the aging population. Recent modelling suggests that there will be a 120% increase in the number of adults aged over 65 years by 2050^6^, bringing the total to 1.55 billion people. However, many of the associations and outcomes attributed to MASLD have been studied in middle-aged adults with a relative dearth of studies in older persons. While we have previously shown that MASLD (defined using the Fatty Liver Index [FLI]^7^) is associated with prevalent frailty^8^ as well as incident physical disability^9^ and atrial fibrillation (AF) (though not major adverse cardiovascular events [MACE] or all-cause mortality)^10^, other large epidemiological studies relying on available laboratory values and anthropometry may not be able to calculate the FLI.

Of the non-invasive biomarker tools available to rule-in or rule-out hepatic steatosis in large epidemiological studies, the FLI is the best-validated in older adults^11^; others, including the Framingham Steatosis Index (FSI)^12^, Dallas Steatosis Index (DSI)^13^, Hepatic Steatosis Index (HSI)^14^, Lipid Accumulation Product (LAP)^15^, Visceral Adiposity Index (VAI)^16^ and the ZJU index (ZJU)^17^ have been predominantly used in middle-aged adults. Similarly, while various studies have compared some of these indices^15,18,19^, these were also predominantly in middle-aged adults. This is of particular importance when considering their utility for older adults, especially given the known decrease in alanine aminotransferase (ALT) that occurs with age^20,21^ and the role of ALT in some of these scores.

Furthermore, there is little data directly comparing the ability of these scores to identify participants at risk of clinically relevant outcomes. This is significant given the practical and economic limitations of relying on histology or imaging to identify MASLD in epidemiological studies, leading many to rely on non-invasive tests for case-identification^22^. Additionally, the diagnosis of metabolic dysfunction-associated fatty liver disease (MAFLD)^23^ explicitly allows for the use of serum biomarkers and scores such as FLI as an alternative method for the diagnosis of steatosis^24^, underpinning the relevance of comparing the performance of scores to each other.

Given this, we aimed to assess the relationship between these scores to rule-in and rule-out MASLD compared with the FLI in older adults, as well as assess the correlations between the scores. Additionally, we aimed to assess which score(s) performed best in stratifying older adults for important clinical outcomes commonly associated with MASLD including MACE, AF, and persistent physical disability.

## Results

### Baseline characteristics and relationships between SLD scores

**Fig. 1:**
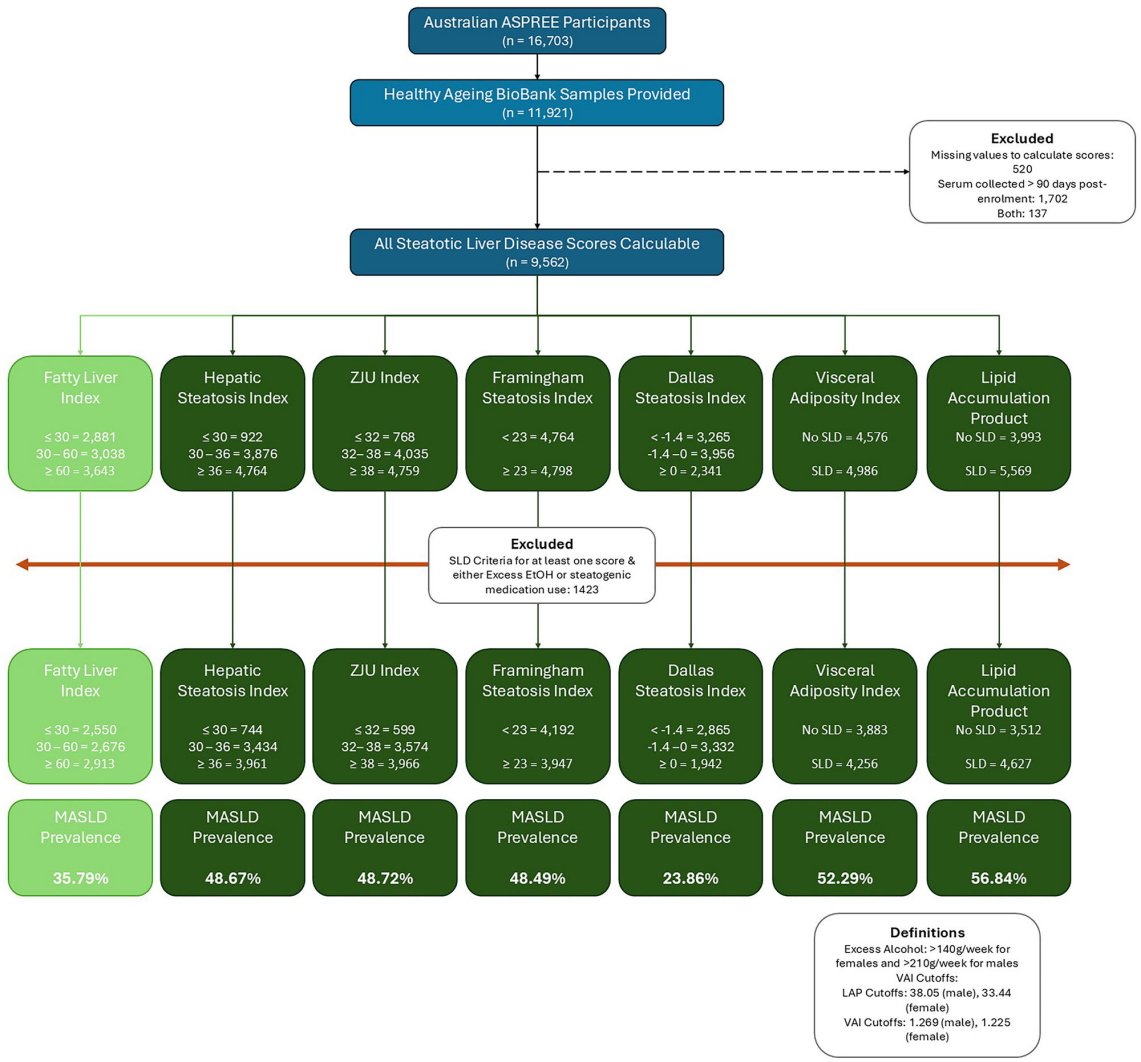
Participant flow diagram.

Of the 16,703 Australian participants, 11,914 provided serum for the Healthy Ageing Biobank. After excluding those with missing components required for the calculation of the SLD scores or those in whom serum was collected more than 90 days from enrolment in ASPREE, 9562 had calculable scores. Subsequently, those with scores above the previously defined cut-offs for MASLD (Supplementary Table 3), who were drinking excess alcohol and/or taking steatogenic medications were excluded, leaving 8,139 for the final analysis (Fig. 1).

Baseline characteristics of the study participants can be seen in Table 1. The overall prevalence of FLI-defined MASLD was 35.8%. Those with MASLD defined by the FLI were more likely to be male, be younger, have cardiometabolic comorbidities (including type 2 diabetes mellitus [T2DM] and chronic kidney disease [CKD]), be overweight/obese, and have elevated liver enzymes. Of the alternative SLD scores, all but the DSI led to classifying more participants as having MASLD than the FLI (48.5% – 56.8% vs 35.8%); the DSI classified only 23.9% as MASLD (Fig. 1). When considering the correlation between the categorical scores and the FLI categories, the Spearman’s Rho is strongest for the FSI (0.721) and DSI (0.727), and weakest for the VAI (0.472) (Supplementary Table 3). Results are similar when using the scores as continuous variables (Supplementary Table 4). Similarly, when comparing the AUROCs of the individual scores against FLI-defined MASLD vs FLI < 60, the FSI performed the best (0.933), followed by ZJU (0.921), LAP (0.921), DSI (0.912), HSI (0.899), and the VAI (0.795).

**Table 1:** Baseline demographics and health conditions

	No MASLD (FLI < 30) (*n* = 2550)	Indeterminate (FLI 30–60) (*n* = 2676)	MASLD (FLI ≥ 60) (*n* = 2913)	Total (*n* = 8139)	*p*
Age (years, median [IQR])	74.2 (71.7–78.0)	74.0 (71.7–77.7)	73.6 (71.5–76.7)	73.9 (71.7–77.4)	<0.001^a^
Sex (male, *n* [%])	788 (30.9%)	1370 (51.2%)	1504 (51.6)	3662 (45.0%)	<0.001^b^
Self-Described Ethnicity					0.034^b^
Caucasian (*n*, %)	2500 (98.0%)	2642 (98.7%)	2884 (99.0%)	8026 (98.6%)	
Black (*n*, %)	1 (0.0%)	2 (0.1%)	1 (0.0%)	4 (0.0%)	
Asian (*n*, %)	32 (1.3%)	16 (0.6%)	14 (0.5%)	62 (0.8%)	
Other (*n*, %)	17 (0.7%)	16 (0.6%)	14 (0.5%)	47 (0.6%)	
BMI (kg/m^2^, mean ± SD)	24.1 ± 2.3	27.3 ± 2.2	32.0 ± 4.0	28.0 ± 4.4	<0.001^c^
BMI Categories					<0.001^b^
Underweight (*n*, %)	31 (1.2%)	0 (0.0%)	0 (0.0%)	31 (0.4%)	
Healthy weight (*n*, %)	1639 (64.3%)	350 (13.1%)	31 (1.1%)	2020 (24.8%)	
Overweight (*n*, %)	856 (33.6%)	2000 (74.7%)	949 (32.6%)	3805 (46.8%)	
Obese (*n*, %)	24 (0.9%)	326 (12.2%)	1933 (66.4%)	2283 (28.1%)	
Abdominal Circumference (cm, mean ± SD)	84.8 ± 7.6	96.1 ± 6.4	107.6 ± 9.1	96.7 ± 12.2	<0.001^c^
T2DM (*n*, [%])	93 (3.6%)	174 (6.5%)	488 (16.8%)	755 (9.3%)	<0.001^b^
Hypertension (*n*, [%])	1408 (55.2%)	1584 (59.2%)	1918 (65.8%)	4910 (60.3%)	<0.001^b^
CKD (*n*, [%])	548 (22.8%)	595 (23.7%)	823 (30.1%)	1966 (25.7%)	<0.001^b^
Laboratory values					
GGT (U/L, median [IQR])	16 (13–21)	20 (16–28)	28 (20–42)	21 (16–30)	<0.001^a^
ALT (U/L, median [IQR])	16 (13–20)	18 (14–22)	21 (16–27)	18 (14–23)	<0.001^a^
AST (U/L, median [IQR])	21 (18–24)	20 (17–23)	21 (18–25)	20 (18–24)	<0.001^a^
Glucose (mmol/L, mean ± SD)	5.2 ± 0.7	5.4 ± 0.8	5.8 ± 1.2	5.5 ± 1.0	<0.001^c^
Cholesterol (mmol/L, mean ± SD)	5.3 ± 1.0	5.3 ± 1.0	5.2 ± 1.0	5.2 ± 1.0	<0.001^c^
HDL-C (mmol/L, mean ± SD)	1.8 ± 0.5	1.5 ± 0.4	1.4 ± 0.4	1.6 ± 0.5	0.024^c^
Triglycerides (mmol/L, mean ± SD)	0.9 ± 0.3	1.2 ± 0.5	1.7 ± 0.8	1.3 ± 0.6	<0.001^c^
Lipid accumulation Product					<0.001^b^
No MASLD (*n*, %)	2257 (88.5%)	1097 (41.0%)	158 (5.4%)	3512 (43.2%)	
MASLD (*n*, %)	293 (11.5%)	1579 (59.0%)	2755 (94.6%)	4627 (56.8%)	
Hepatic steatosis index					<0.001^b^
No MASLD (*n*, %)	669 (26.2%)	69 (2.6%)	6 (0.2%)	744 (9.1%)	
Indeterminate (*n*, %)	1647 (64.6%)	1445 (54.0%)	342 (11.7%)	3434 (42.2%)	
MASLD (*n*, %)	234 (9.2%)	1162 (43.4%)	2565 (88.1%)	3961 (48.7%)	
ZJU index					<0.001^b^
No MASLD (*n*, %)	581 (22.8%)	18 (0.7%)	0 (0.0%)	599 (7.4%)	
Indeterminate (*n*, %)	1783 (69.9%)	1516 (56.7%)	275 (9.4%)	3574 (43.9%)	
MASLD (*n*, %)	186 (7.3%)	1142 (42.7%)	2638 (90.6%)	3966 (48.7%)	
Framingham steatosis index					<0.001^b^
No MASLD (*n*, %)	2442 (95.8%)	1519 (56.8%)	231 (7.9%)	4192 (51.5%)	
MASLD (*n*, %)	108 (4.2%)	1157 (43.2%)	2682 (92.1%)	3947 (48.5%)	
Dallas steatosis index					<0.001^b^
No MASLD (*n*, %)	2022 (79.3%)	764 (28.6%)	79 (2.7%)	2865 (35.2%)	
Indeterminate (*n*, %)	519 (20.4%)	1671 (62.4%)	1142 (39.2%)	3332 (40.9%)	
MASLD (*n*, %)	9 (0.4%)	241 (9.0%)	1692 (58.1%)	1942 (23.9%)	
Visceral adiposity index					<0.001^b^
No MASLD (*n*, %)	1978 (77.6%)	1323 (49.4%)	582 (20.0%)	3883 (47.7%)	
MASLD (*n*, %)	572 (22.4%)	1353 (50.6%)	2331 (80.0%)	4256 (52.3%)	

*BMI* Body Mass Index, Categories: Underweight (<20 kg/m^2^), Healthy weight (White: 20–25 kgm^2^, Asian: 20–23 kg/m^2^), Overweight (White: 25–30 kg/m^2^; Asian 23–25 kg/m^2^), Obese (White: ≥30 kg/m^2^; Asian ≥25 kg/m^2^), *CKD* Chronic Kidney Disease was defined as eGFR <60 ml/kg/m^2^ and/or an elevated urinary albumin-creatinine ratio (>35 mg/g for females or >25 mg/g for males)^37^, *GGT* gamma glutamyltransferase, *ALT* alanine aminotransferase, *AST* aspartate aminotransferase, *HDL-C* high density lipoprotein cholesterol.

^a^Kruskall–Wallis H Test.

^b^Chi squared test.

^c^One way ANOVA.

When subclassifying the validated tools as MASLD vs no-MASLD (by excluding the indeterminate group in non-binary cut-offs), the DSI had the highest percentage of participants correctly classified (97.7%), followed by the FSI (93.8%) (Table 2). The HSI (99.8%) and ZJU (100.0%) were the most sensitive, and the DSI (99.6%) and FSI (95.8%) were the most specific (Table 2). When re-classifying the indeterminate group to no-MASLD, results were similar (Supplementary Table 5). The DSI (81.9%) and FSI (81.6%) correctly classified the most, and the DSI was the most specific (95.2%). However, the sensitivity of the DSI suffered, falling to 58.1% (Supplementary Table 5).

**Table 2:** Classification Table when excluding indeterminate MASLD score values, using FLI < 30 (no MASLD) and FLI ≥ 60 (MASLD) as the comparator

	Correctly classified as FLI-identified MASLD	Sensitivity	Specificity	Positive predictive value	Negative predictive value
Lipid accumulation product	91.74%	94.58%	88.51%	90.39%	93.46%
Hepatic steatosis index	93.09%	99.77%	74.09%	91.64%	99.11%
ZJU index	94.54%	100.00%	75.75%	93.41%	100.00%
Framingham steatosis index	93.79%	92.07%	95.76%	96.13%	91.36%
Dallas steatosis index	97.69%	95.54%	99.56%	99.47%	96.24%
Visceral adiposity index	78.88%	80.02%	77.57%	80.30%	77.27%

### Associations between MASLD scores and outcomes

When generating Cox proportional hazards models (adjusted for age and sex) (Table 3), none of the MASLD scores was associated with an increased risk of mortality. Indeed, the MASLD defined by the ZJU or HSI are both inversely associated with mortality (HR 0.71 [95% CI 0.59–0.85] and HR 0.72 [95% CI 0.60–0.86], respectively).

**Table 3:** Cox proportional hazard models (adjusted for age and sex)

MASLD scores	All-cause mortality	MACE	Atrial fibrillation	Persistent physical disability
FLI (HR [95% CI]) <30 (No MASLD)				
30–60	0.92 (0.79–1.06)	1.19 (0.97–1.46)	1.16 (0.91–1.50)	1.14 (0.90–1.44)
≥60 (MASLD)	1.10 (0.96–1.27)	**1.36** (1.11–1.66)	**1.48** (1.16–1.88)	**2.12** (1.72–2.62)
LAP (HR [95% CI])				
No MASLD MASLD	1.06 (0.94–1.19)	**1.41** (1.20–1.66)	1.12 (0.93–1.36)	**1.60** (1.34–1.91)
HSI (HR [95% CI]) <30 (No MASLD)				
30–36	**0.66** (0.55–0.78)	0.83 (0.63–1.08)	0.94 (0.67–1.32)	0.71 (0.53–0.96)
≥36 (MASLD)	**0.72** (0.60–0.86)	1.05 (0.80–1.37)	1.24 (0.88–1.73)	1.32 (0.99–1.76)
ZJU (HR [95% CI]) <32 (No MASLD)				
32–38	**0.63** (0.53–0.76)	0.96 (0.71–1.30)	0.83 (0.59–1.18)	**0.65** (0.47–0.90)
≥38 (MASLD)	**0.71** (0.59–0.85)	1.18 (0.87–1.60)	1.08 (0.76–1.53)	1.26 (0.92–1.71)
FSI (HR [95% CI])				
No MASLD MASLD	0.99 (0.88–1.11)	**1.24** (1.06–1.46)	**1.25** (1.03–1.51)	**1.74** (1.47–2.07)
DSI (HR [95% CI]) < −1.4 (no MASLD)				
−1.4–0	0.93 (0.81–1.06)	1.06 (0.88–1.27)	1.14 (0.92–1.41)	**1.28** (1.05–1.57)
≥0 (MASLD)	1.04 (0.89–1.21)	1.21 (0.98–1.49)	1.13 (0.87–1.46)	**1.90** (1.53–2.37)
VAI (HR [95% CI]) No MASLD				
MASLD	1.08 (0.87–1.22)	**1.49** (1.27–1.75)	0.94 (0.78–1.14)	**1.41** (1.18–1.67)

Bold values note statistical significance.

When considering cardiovascular outcomes, the LAP (HR 1.41 [95% CI 1.20–1.66]) and VAI (HR 1.49 [95% CI 1.27–1.75]) were strongly associated with incident MACE. MASLD identified by the FSI was also associated with MACE, though the relationship was weaker than MASLD defined by the FLI (FSI HR 1.24 [95% CI 1.06–1.46] and FLI HR 1.36 [95% CI 1.11–1.66], both compared to no steatosis). Interestingly, there was no relationship between MASLD determined by the HSI, ZJU or DSI with MACE. For incident AF, however, only the FSI (HR 1.25 [95% CI 1.03–1.51]) and FLI (HR 1.48 [95% CI 1.16–1.88]) were predictive; all the other scores were not associated with AF.

Finally, when considering persistent physical disability, the DSI had the strongest relationship outside of the FLI (HR 1.90 [95% CI 1.53–2.37]). The FSI (HR 1.74 [95% CI 1.47–2.07]), LAP (HR 1.60 [95% CI 1.34–1.91]), and VAI (HR 1.41 [95% CI 1.18–1.67]) were also associated with physical disability, although the ZJU and HSI were not.

When re-classifying the scores from tertiles to binary scores (where indeterminate is classified as no-MASLD), the overall performance in terms of predicting outcomes varies for each score (Supplementary Table 6). The HSI and ZJU have an increase in utility, and are now associated with MACE, AF, and physical disability while no longer being inversely associated with all-cause mortality. The performance of the DSI worsened – there remained no association between the DSI and MACE or AF, and there’s a reduced hazard for incident persistent physical disability using this binary cut-off (HR 1.66 [95% CI 1.38–2.00]) (Supplementary Table 6). Using the <60 and ≥60 (MASLD) cut-offs for the FLI led to a weak association with mortality (HR 1.15 [95% CI 1.02–1.30]), but otherwise reduced hazard ratios for the other outcomes of interest (Supplementary Table 6).

## Discussion

MASLD is a problem of not just the middle-aged but also of older adults^8,25^, where it is associated with MACE, AF, and persistent physical disability^9^ (though not mortality)^26^. A variety of epidemiological tools exist to rule-in and rule-out MASLD, though their validity in older adults has not been well studied. While the FLI has been previously validated in a large cohort of older adults^11^, and incorporated as a validated tool to define fatty liver in the MAFLD diagnostic algorithm^23,24^, not all pre-existing cohort studies will have each of the required variables to calculate the FLI (triglycerides, body mass index [BMI], abdominal circumference, and gamma-glutamyl transferase [GGT]), and so the use of other non-invasive tests relying on different anthropometric and laboratory parameters may be required. In particular, abdominal circumference isn’t routinely assessed and recorded in inpatient and outpatient clinical practice, and some epidemiological studies will not have a complete panel of liver function tests performed in relation to GGT levels. As such, we aimed to understand the relationship between MASLD as identified using the FLI and other non-invasive tests used to identify MASLD to assist with determining their relative accuracies and their ability to predict clinically relevant outcomes in older adults. The key findings of this study are that the FSI^12^ and DSI^13^ are best at correctly categorizing older adults as MASLD vs no-MASLD, and the FSI is better than the DSI at identifying patients at risk of MACE, AF, and persistent physical disability, supporting its use in other epidemiological studies of older adults where the FLI is unable to be calculated.

Various community-based studies have been performed utilizing either radiology^25^ or non-invasive scores^8,27,28^ (such as the FLI) to estimate the prevalence of MASLD in older adults. While these estimates vary based on the year the data were collected (given the increasing rates of MASLD over time), the population group, and identification tool used, estimates tend to range from 30 to 40%. In our study using FLI as the standard, the prevalence of MASLD was 35.6%, consistent with these results. However, all other scores evaluated, except the DSI, estimated a MASLD prevalence of 48.5–56.8%, likely representing a mischaracterization or overestimate of MASLD prevalence in this group. It’s possible that reclassifying the previously specified cut-off values for these scores in older persons may improve their accuracy and avoid overestimating the prevalence of MASLD; this would be a useful area of future study. Interestingly, the DSI provided a much lower estimate of prevalent MASLD – only 23.9%. The cause of this is lower prevalence estimate from the DSI is uncertain, though it may relate to the specific categorical variable cut-offs used in the score. The population examined here is older than that investigated in the derivation cohort of the Dallas Heart Study^13^ (with consequently lower transaminase levels^20^), potentially skewing the DSI scores to lower values and thus below the pre-specified MASLD cut-off.

Furthermore, when considering the ‘indeterminate’ groups, those categorized as such by the HSI (42.2%), ZJU (43.9%) and DSI (40.9%) were all larger than the FLI (32.9%). These rates of ‘indeterminate’ were lower for the HSI and ZJU than in the original derivation and validation papers^14,17^, though they were higher in the DSI group (41% vs 32%)^13^. However, the ‘no MASLD’ groups were much smaller in our study compared to the original papers for both the HSI^14^ (9.1% vs 23.5%) and ZJU^17^ (7.4% vs 25.3%). For the HSI and ZJU, this is likely reflective of the different distribution of BMI in our cohort vs the derivation cohorts. For the DSI – which relies on granular gradations of ALT level – it’s possible this is influenced by the relatively lower median ALT values in older adults than those seen in the middle-aged derivation cohort.

When indeterminate values were excluded, the FSI and DSI were best at correctly classifying patients as MASLD vs no-MASLD (93.8% and 97.7% respectively). Both have sensitivities and specificities of over 90% supporting their utility in identifying MASLD compared to the FLI. Interestingly, while the sensitivities of the HSI (99.8%) and ZJU (100.0%) were excellent, their specificities were markedly worse. This is likely reflective of the populations from which they were derived. Both the HSI and ZJU were created and validated in Asian populations and rely heavily on the BMI in their score; given the relationship between BMI and metabolic diseases is different in Asian compared to non-Asian populations it’s possible that the cut-offs generated by these scores aren’t optimized for the predominantly White population in this study. Of the scores used for purposes other than pure MASLD identification, the VAI performed poorly overall in terms of correct classification of MASLD (78.9%), with relatively lower sensitivity (80.0%) and specificity (77.6%) than the LAP (94.6% and 88.5% respectively).

When considering the relationship between score-defined MASLD and important outcomes, our findings are consistent with previous research showing that SLD is not strongly related to mortality in older adults^26,28^. Most of the scores evaluated here were not associated with all-cause mortality when adjusting for age and sex. Interestingly, the ZJU (HR 0.72 [95% CI 0.60–0.86]) and HSI (HR 0.71 [95% CI 0.59–0.85]) were inversely associated with mortality in this population of older adults. While the cause of this is uncertain, it’s likely due to a combination of factors. Both the HSI and ZJU are reliant on BMI. There is a known U-shaped association between BMI and mortality in older adults^29^, suggesting that those with low scores may be at increased risk of mortality due to a low BMI.

In contrast, SLD is thought to be associated with MACE and potentially AF in older adults, including in an analysis of the ASPREE population. The LAP and VAI were most strongly associated with MACE (HR 1.36 [95% CI 1.11–1.66] and HR 1.49 [95% CI 1.27–1.75] respectively), potentially reflecting their initial derivation as markers of cardiovascular disease risk^16,30^. Of the scores developed specifically for the identification of MASLD, the FSI was associated with an increased risk of MACE (HR 1.24 [95% CI 1.06–1.46]), though the HSI, ZJU, and DSI were not. Similarly, while the FSI was associated with incident AF (HR 1.25 [95% CI 1.03–1.51]), no other markers apart from the FLI had a relationship with AF.

Of the non-FLI markers, MASLD as defined by the DSI had the strongest relationship with incident persistent physical disability (HR 1.90 [95% CI 1.53–2.37]), followed by the FSI (HR 1.74 [95% CI 1.47–2.07]). There was no relationship between the HSI or ZJU and physical disability. The LAP and VAI both performed worse than the FSI and DSI.

Our study has numerous strengths, including the large sample size, rigorous data collection during enrolment and follow-up, and robust end-point ascertainment. However, some limitations should be discussed. The use of the FLI as the ‘gold standard’ in this study is an inherent limitation – while it has been validated in an older Caucasian population similar to ours^11^, it is not the gold standard for the diagnosis of MASLD. Future work comparing the scores to histology or MRI-defined steatosis would be valuable. Additionally, the ASPREE population was a relatively healthy community-dwelling group at enrolment – care should be taken when extrapolating these findings to different population groups. MASLD exclusion criteria were partly derived from alcohol intake self-report, which may not be reliable. Significant miscategorisation of alcohol intake in the MASLD group may impact some of the biochemical parameters differently to that seen in low- or no-alcohol MASLD, potentially reducing the accuracy of the non-invasive scores. However, due to the conservative calculation used to identify alcohol intake above the MASLD threshold (where the higher end of every estimated intake range for both amount and frequency was selected), it’s unlikely that a significant number of participants actually meeting MetALD criteria would have been misclassified as MASLD.

In conclusion, this large community-based cohort study of relatively healthy older persons, we have shown significant variation in the prevalence of MASLD when defined according to different non-invasive biomarker tests. Additionally, we have shown that the FSI and DSI are the most accurate scores for identifying MASLD when using the FLI as the gold standard, though consideration of adjusting cut-offs in an older population may be warranted. Finally, we have shown that – consistent with the FLI – the FSI is associated with incident MACE, AF, and persistent physical disability, lending support to its use in epidemiological studies of older persons where the FLI is unable to be calculated.

## Methods

### Study population

We performed a retrospective analysis of all Australian participants included in the ASPirin in Reducing Events in the Elderly (ASPREE) randomised trial and ASPREE-eXTension (ASPREE-XT) Cohort Study; the study designs and findings from the main trial have been previously published in detail^31–34^. In brief, between 2010 and 2014 ASPREE recruited 16,703 Australian participants via their usual primary care providers. These participants were aged 70+ years, had a life expectancy of at least five years, and were free from baseline dementia, established or previous cardiovascular disease (including atrial fibrillation), and were functionally independent. Of note, participants weren’t excluded because of underlying liver disease unless that disease was expected to limit their life expectancy to less than five years or was associated with a high risk of bleeding. Participants were followed through annual in-person visits, medical record reviews, and between-visit telephone contact. All participants provided written informed consent. The ASPREE, ASPREE-XT, and ASPREE Healthy Ageing Biobank studies were approved by local ethics committees. ASPREE and ASPREE-XT are registered on ClinicalTrials.gov (NCT01038583), and the ASPREE trial is registered on the International Standard Randomised Controlled Trial Number Registry (ISRCTN83772183).

The initial ASPREE trial was approved by the Monash University Human Research Ethics Committee (MUHREC) (IRB00002519; ethics #2006/745MC) and other allied institution ethics committees. In Australia, the Alfred Hospital Ethics Committee (ethics #HREC/17/Alfred/198) oversees the ASPREE-XT project as the primary site approver. The ASPREE clinical trial is registered on ClinicalTrials.gov (NCT01038583) and the International Standard Randomised Controlled Trial Number Registry (ISRCTN83772183). ASPREE-XT is registered with ClinicalTrials.gov (NCT01038583).

### Participant assessment & laboratory data

At baseline (and during follow-up), in-person interviews and assessments collated information on lifestyle, social, and medical history; self-described ethnicity; prescription medications were recorded by Anatomical Therapeutic Code (ATC); and laboratory parameters were requested and stored (including fasting glucose and a fasting lipid profile performed annually at local pathology laboratories). Anthropometry and physical markers were also collected, including weight (with excess clothing removed on calibrated scales), height, and abdominal circumference. Blood pressure was measured in the seated position following 5 min of rest with two measurements taken 1 min apart. Additionally, Australian participants were offered the opportunity to participate in the ASPREE Healthy Aging Biobank sub-study, where non-fasting serum was collected within the first year of the ASPREE study for storage and subsequently used for biochemical analysis to determine the gamma-glutamyl transferase (GGT), ALT, and aspartate aminotransferase (AST)^35^. The analysis of the Healthy Ageing Biobank serum was performed centrally using an Abbott Alinity ci series analyser (Abbott Diagnostics).

### Chronic conditions and baseline characteristics

For the purposes of this study, participants were stratified as current, former, or never alcohol drinkers. Excess alcohol intake was defined as males >210 g/week and females >140 g/week^36^. T2DM was defined as one or more of: self- or physician-reported T2DM, a fasting glucose ≥7 mmol/L, and/or the prescription of hypoglycaemic medication(s). HbA1c was not measured as part of either the ASPREE study or the Healthy Aging Biobank sub-study. The criteria of metabolic dysfunction that are required for the definition of MASLD were used as previously published^1^ – one or more of: BMI ≥ 25 kg/m^2^ (or ≥23 kg/m^2^ in those of Asian ethnicity), elevated abdominal circumference (using sex-specific cut-offs – male ≥94 cm, female ≥80 cm), T2DM (as defined above), a fasting blood glucose ≥5.6 mmol/L, blood pressure ≥130/85 mmHg, the prescription of an antihypertensive, fasting triglycerides ≥1.70 mmol/L, fasting High Density Lipoprotein (HDL) < 1.0 mmol/L (for males) or <1.3 mmol/L (for females), or lipid lowering therapy. Chronic kidney disease (CKD) was defined as eGFR <60 ml/kg/m^2^ and/or an elevated urinary albumin-creatinine ratio (>35 mg/g for females or >25 mg/g for males)^37^.

### Defining hepatic steatosis

A variety of composite scores based on medical history, biochemistry, and anthropometry were collected and calculated as previously described^7,11–17^ (Supplementary Table 1). Participants were classified as MASLD if they met the pre-determined score thresholds to ‘rule-in’ steatotic liver disease and concomitantly met usual MASLD criteria (including at least one feature of metabolic dysfunction; the absence of excess alcohol; and the absence of steatogenic medications including corticosteroids, tamoxifen, methotrexate, and/or amiodarone).

### Defining outcomes

MACE was defined (as per the original ASPREE publication^38^ and based on World Health Organization criteria) as a composite of fatal coronary heart disease (excluding death from heart failure), nonfatal acute myocardial infarction (AMI), or fatal or nonfatal ischemic stroke^38^. Incident MACE was adjudicated from supporting medical documentation as previously described^31^. Source information from hospitals/medical centers, treating physicians, death certificates, medical records, hospital information obtained from the next of kin or other family members where relevant was collected, sent to the ASPREE Data Management Center and presented to adjudicators. Each case was independently reviewed by two clinical experts, with a third adjudicator resolving discordance. The identification of incident AF occurred as part of a separate ASPREE sub-study^39^ during the trial period only and was based on ‘triggers’ for chart reviews of medication, correspondence, and ECGs (if available). The use of the AF endpoint in ASPREE MASLD studies has been previously described in detail^10^.

Persistent physical disability was defined as the loss of capacity to perform independently at least 1 of 6 basic activities of daily living (ADLs) including: walking across a room, transferring from bed or chair, toileting, bathing, dressing and eating, as described^32^. Alternatively, assessment confirming the need or eligibility for admission to a nursing care facility for a physical disability was also considered persistent physical disability. These data were collected every six months, and persistent loss of function was determined by the loss of capacity to perform the same ADL for at least six months. Time to event was the time the loss of the ADL was first reported or the first noted assessment of the need for residential care admission^40^.

### Statistics

Baseline data were compared using a one-way ANOVA, Student’s *t* test, Kruskal–Wallis test, or Chi-squared test as appropriate. The cut-offs used to define ‘rule-in’ and ‘rule-out’ criteria for MASLD were based on those used in previous MASLD publications^7,12–15,17^. Given the FLI is best validated in older adults, it was used as the ‘gold standard’ to which other scores were compared to determine their relative sensitivity and specificity for older adults asthere is no available radiological or histological data in ASPREE to serve as alternative comparators for hepatic steatosis. The components and calculations for the non-invasive scores can be seen in Supplementary Tables 1 and 2. The correlation between ordinal categories of the scores (either rule-out/rule-in, or rule-out/indeterminate/rule-in) were calculated using a Spearman’s Rank-Order Correlation. Areas under the receiver operating curve (AUROCs) were determined, using FLI ≥ 60 vs FLI < 60 to rule-in/rule-out MASLD. Finally, Cox proportional hazard regression models adjusted for age and sex were utilized to evaluate the relationship between MASLD vs no-MASLD (defined by each score) and MACE, AF, and persistent physical disability. Sensitivity analyses were performed where, for scores in which ‘indeterminate’ scores were possible, these ‘indeterminate’ scores were classified as ‘no-MASLD’. A *p* value of <0.05 was considered statistically significant. Statistical analyses were performed using Stata software v17.0 (StataCorp LLC, College Station, TX).

## Supplementary information

Supplementary Tables

## Data Availability

The datasets used and/or analysed for this publication are available via the ASPREE Principal Investigators. Requests for data access can be directed to aspree.ams@monash.edu.
