# Supplementary material for: Scores to predict steatotic liver disease – correlates and outcomes in older adults
Source: NPJ Gut Liver. 2025 Apr 19;2(1):9. doi: 10.1038/s44355-025-00021-3 (PMC12009214; doi:10.1038/s44355-025-00021-3)
Supplement: Supplementary file 1 — Supplementary Tables [file 44355_2025_21_MOESM1_ESM.pdf]

**Supplementary Tables**

| Score Components | FLI | FSI | DSI | HSI | ZJU | VAI | LAP |
|---|---|---|---|---|---|---|---|
| BMI (kg/m$^2$) | ✓ | ✓ | ✓ | ✓ | ✓ | ✓ | |
| Abdominal Circumference (cm) | ✓ | | | | | ✓ | ✓ |
| GGT (U/L) | ✓ | | | | | | |
| Triglycerides | ✓ | ✓ | ✓ | | ✓ | ✓ | ✓ |
| ALT (U/L) | | ✓ | ✓ | ✓ | ✓ | | |
| AST (U/L) | | ✓ | | ✓ | ✓ | | |
| Age | | ✓ | ✓ | | | | |
| Sex | | ✓ | ✓ | ✓ | ✓ | ✓ | ✓ |
| Hypertension | | ✓ | ✓ | | | | |
| Diabetes | | ✓ | ✓ | ✓ | | | |
| Serum Glucose | | | ✓ | | ✓ | | |
| Ethnicity | | | ✓ | | | | |
| HDL Cholesterol | | | | | | ✓ | |

Supplementary Table 1 – Score Components

| Scores | Calculation Formula | |
|---|---|---|
| FLI | $e^y / (1 + e^y)$ | $y = 0.953 \times \ln(Tg[mg\ dL^{-1}]) + 0.139 \times BMI + 0.718 \times \ln(GGT) + 0.053 \times AC - 15.745$ |
| FSI | $-7.981 + 0.011 \times age - 0.146 \times sex(female = 1, male = 0) + 0.173 \times BMI + 0.007 \times Tg(mg\ dL^{-1}) + 0.593 \times hypertension(yes = 1, no = 0) + 0.789 \times T2DM(yes = 1, no = 0) + 1.1 \times (\frac{ALT}{AST} \geq 1.33\ [yes = 1, no = 0])$ | |
| DSI | $-9.4 + 0.316\ (if\ Age\ \geq 50\ and\ Female) + 2.4\ (if\ known\ T2DM) + 0.02 \times (0\ if\ T2DM, else\ Glucose\ [mg\ dL^{-1}]) + 0.3\ (if\ hypertensive) + 0.5\ (if\ Hispanic/Asian/Other\ Race) + ln(Tg\ [mg\ dL^{-1}]) + 0.4\ (if\ ALT\ 13.5 - 19.49) + 1.1\ (if\ ALT\ 19.5 - 40) + 1.5\ (if\ ALT > 40) + 0.7\ (if\ not\ black\ and\ BMI\ 25 - 27.49) + 1.4\ (if\ not\ black\ and\ BMI\ 27.5 - 34.9) + 1.9\ (if\ not\ black\ and\ BMI\ 35 - 37.49) + 2.6\ (if\ not\ black\ and\ BMI > 37.5) - 0.2\ (if\ black\ and\ BMI\ 25 - 27.49) + 0.8\ (if\ black\ and\ BMI\ 27.5 - 34.9) + 0.8\ (if\ black\ and\ BMI\ 35 - 37.49) + 1.8\ (if\ black\ and\ BMI > 37.5)$ | |
| HSI | $8 \times \frac{ALT}{AST} + BMI + (2\ if\ T2DM) + (2\ if\ Female)$ | |
| ZJU | $BMI + Fasting\ Glucose\ (mmol\ L^{-1}) + Tg\ (mmol\ L^{-1}) + 3 \times \frac{ALT}{AST} + (2\ if\ Female)$ | |
| VAI | Males $\frac{AC\ [cm]}{39.68 + (1.89 \times BMI)} \times \frac{Tg\ (mmol\ L^{-1})}{1.03} \times \frac{1.31}{HDL(mmol\ L^{-1})}$ | Females $\frac{AC\ [cm]}{36.58 + (1.89 \times BMI)} \times \frac{Tg\ (mmol\ L^{-1})}{0.81} \times \frac{1.52}{HDL(mmol\ L^{-1})}$ |
| LAP | Males $(AC\ [cm] - 65) \times Tg\ (mmol\ L^{-1})$ | Females $(AC\ [cm] - 58) \times Tg\ (mmol\ L^{-1})$ |

Supplementary Table 2 – Score Formulae

BMI: Body Mass Index (kg/$m^2$)
AC: Abdominal Circumference
Tg: Serum triglycerides
GGT: gamma glutamyltransferase (U/L)
T2DM: type 2 diabetes mellitus
ALT: alanine aminotransferase (U/L)
AST: aspartate aminotransferase (U/L)

| | FLI Categories | LAP Categories | HSI Categories | ZJU Categories | FSI Categories | DSI Categories | VAI Categories |
|---|---|---|---|---|---|---|---|
| FLI Categories | 1.0 | | | | | | |
| LAP Categories | 0.6828 | 1.0 | | | | | |
| HSI Categories | 0.6693 | 0.4950 | 1.0 | | | | |
| ZJU Categories | 0.7039 | 0.5552 | 0.8612 | 1.0 | | | |
| FSI Categories | 0.7214 | 0.5863 | 0.6517 | 0.6979 | 1.0 | | |
| DSI Categories | 0.7270 | 0.6169 | 0.7013 | 0.7279 | 0.7053 | 1.0 | |
| VAI Categories | 0.4719 | 0.6931 | 0.3082 | 0.3689 | 0.4366 | 0.4833 | 1.0 |

Supplementary Table 3 – Spearman's coefficients for SLD scores (using categorical cut-offs)

| | FLI | LAP | HSI | ZJU | FSI | DSI | VAI |
|---|---|---|---|---|---|---|---|
| FLI | 1.0 | | | | | | |
| LAP | 0.8583 | 1.0 | | | | | |
| HSI | 0.7949 | 0.6244 | 1.0 | | | | |
| ZJU | 0.8360 | 0.7090 | 0.9611 | 1.0 | | | |
| FSI | 0.8647 | 0.7790 | 0.8245 | 0.8652 | 1.0 | | |
| DSI | 0.8230 | 0.7728 | 0.8319 | 0.8612 | 0.8551 | 1.0 | |
| VAI | 0.5918 | 0.8562 | 0.3958 | 0.4837 | 0.5773 | 0.6140 | 1.0 |

Supplementary Table 4 – Spearman's coefficients for SLD scores (as continuous variables)

| | Correctly Classified as FLI-identified MASLD | Sensitivity | Specificity | Positive Predictive Value | Negative Predictive Value |
|---|---|---|---|---|---|
| Lipid Accumulation Product | 75.06% | 94.58% | 64.18% | 59.54% | 95.50% |
| Hepatic Steatosis Index | 78.57% | 88.05% | 73.29% | 64.76% | 91.67% |
| ZJU Index | 80.30% | 90.56% | 74.59% | 66.52% | 93.41% |
| Framingham Steatosis Index | 81.62% | 92.07% | 75.79% | 67.95% | 94.49% |
| Dallas Steatosis Index | 81.93% | 58.08% | 95.22% | 87.13% | 80.30% |
| Visceral Adiposity Index | 69.20% | 80.02% | 63.16% | 54.77% | 85.01% |

Supplementary Table 5 – Classification Table when re-coding indeterminate score values to no MASLD, using FLI < 60 (no MASLD) and FLI ≥ 60 (MASLD) as the comparator.

| MASLD Scores | All-Cause Mortality | MACE | Atrial Fibrillation | Persistent Physical Disability |
|---|---|---|---|---|
| FLI (HR [95% CI]) | | | | |
| < 60 (No MASLD) | | | | |
| ≥ 60 (MASLD) | **1.15** (1.02 – 1.30) | **1.23** (1.04 – 1.44) | **1.36** (1.12 – 1.66) | **1.98** (1.67 – 2.35) |
| LAP (HR [95% CI]) | | | | |
| No MASLD | | | | |
| MASLD | 1.06 (0.94 – 1.19) | **1.41** (1.20 – 1.66) | 1.12 (0.93 – 1.36) | **1.60** (1.34 – 1.91) |
| HSI (HR [95% CI]) | | | | |
| < 36 (No MASLD) | | | | |
| ≥ 36 (MASLD) | 1.00 (0.89 – 1.13) | **1.22** (1.04 – 1.43) | **1.30** (1.07 – 1.58) | **1.73** (1.46 – 2.06) |
| ZJU (HR [95% CI]) | | | | |
| < 38 (No MASLD) | | | | |
| ≥ 38 (MASLD) | 1.03 (0.91 – 1.16) | **1.22** (1.04 – 1.43) | **1.26** (1.04 – 1.53) | **1.79** (1.51 – 2.13) |
| FSI (HR [95% CI]) | | | | |
| No MASLD | | | | |
| MASLD | 0.99 (0.88 – 1.11) | **1.24** (1.06 – 1.46) | **1.25** (1.03 – 1.51) | **1.74** (1.47 – 2.07) |
| DSI (HR [95% CI]) | | | | |
| < 0 (no MASLD) | | | | |
| ≥ 0 (MASLD) | 1.08 (0.93 – 1.24) | 1.17 (0.97 – 1.41) | 1.05 (0.84 – 1.32) | **1.66** (1.38 – 2.00) |
| VAI (HR [95% CI]) | | | | |
| No MASLD | | | | |
| MASLD | 1.08 (0.87 – 1.22) | **1.49** (1.27 – 1.75) | 0.94 (0.78 – 1.14) | **1.41** (1.18 – 1.67) |

Supplementary Table 6 – Cox Proportional Hazard Models (adjusted for age and sex)
